# Characterization of Pseudooxynicotine Amine Oxidase of *Pseudomonas putida* S16 that Is Crucial for Nicotine Degradation

**DOI:** 10.1038/srep17770

**Published:** 2015-12-04

**Authors:** Haiyang Hu, Weiwei Wang, Hongzhi Tang, Ping Xu

**Affiliations:** 1 State Key Laboratory of Microbial Metabolism, and School of Life Sciences & Biotechnology, Shanghai Jiao Tong University, Shanghai 200240, People’s Republic of China; 2 Joint International Research Laboratory of Metabolic & Developmental Sciences, Shanghai Jiao Tong University, Shanghai 200240, People’s Republic of China

## Abstract

Pseudooxynicotine amine oxidase (Pnao) is essential to the pyrrolidine pathway of nicotine degradation of *Pseudomonas putida* strain S16, which is significant for the detoxification of nicotine, through removing the CH_3_NH_2_ group. However, little is known about biochemical mechanism of this enzyme. Here, we characterized its properties and biochemical mechanism. Isotope labeling experiments provided direct evidence that the newly introduced oxygen atom in 3-succinoylsemialdehyde-pyridine is derived from H_2_O, but not from O_2_. Pnao was very stable at temperatures below 50 °C; below this temperature, the enzyme activity increased as temperature rose. Site-directed mutagenesis studies showed that residue 180 is important for its thermal stability. In addition, tungstate may enhance the enzyme activity, which has rarely been reported before. Our findings make a further understanding of the crucial Pnao in nicotine degradation.

Tobacco consumption is not only an important pillar of the national economy of China, but also a leading preventable cause of diseases such as lung cancer^1^,^2^,^3^. Nicotine is the major toxic component of tobacco, and it is capable of crossing biological membranes. When tobacco is burned, nicotine is transformed into tobacco-specific nitrosamines (TSNAs)^4^,^5^,^6^,^7^. One of the most toxic TSNAs, 4-methylnitrosamino-l-(3-pyridyl)-l-butanone (NNK), can be generated from pseudooxynicotine (PN) through nitrosation^8^,^9^. PN is an intermediate product in the nicotine degradation pathway of *Pseudomonas putida* strain S16. Our previous studies showed that strain S16 is a nicotine-metabolizing microorganism that transforms nicotine to 2,5-dihydroxypyridine through *N*-methyl-myosmine, pseudooxynicotine, and 3-succinoylpyridine (SP)^10^,^11^,^12^,^13^. A pseudooxynicotine amine oxidase (Pnao) gene has been identified, and the encoded protein could detoxify PN by removing the CH_3_NH_2_ group;^14^,^15^ when the encoded gene *pnao* was deleted, the pathway of nicotine degradation was blocked, showing this is a critical step for nicotine detoxification by *Pseudomonas*^15^. In previous studies, only the product and the prosthetic group of Pnao were identified^14^. Thus, little is known about Pnao and its mechanism(s) of action.

In this study, His_6_-tagged Pnao was heterologously expressed and purified from *Escherichia coli*, and then characterized. The oxygen atom added to 3-succinoylsemialdehyde-pyridine (SAP) originates from H_2_O; O_2_ is involved in the oxidation of FADH_2_. We also found that Pnao possesses some special properties, such as thermal stability below 50 °C, promotion of enzyme activity by Na_2_WO_4_, and inhibition by Na_2_MoO_4_ and FeCl_3_. The work described here provides a basis for future studies aimed at determining the enzymatic mechanisms of nicotine detoxification.

## Results

### Expression and purification of Pnao

**Figure 1:**
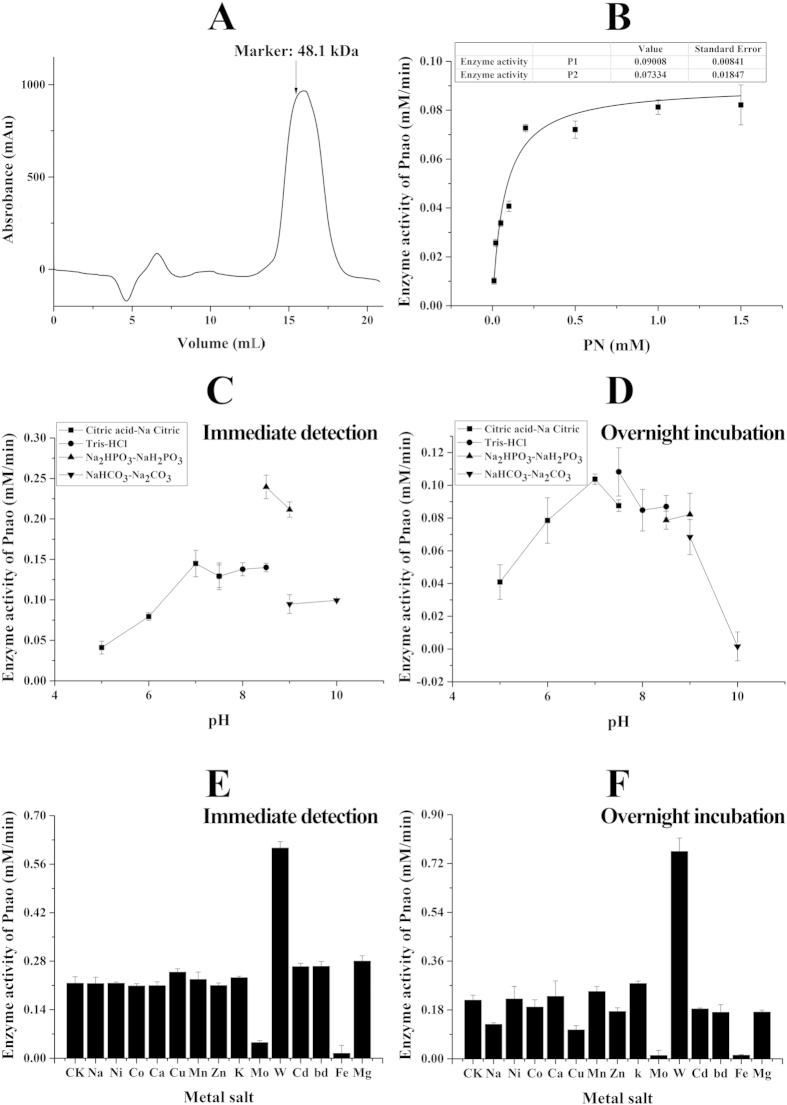
Biochemical characterization by enzymatic assays of Pnao. (**A**) Spectrum curve of Pnao on gel filtration. The column (superdex 200) was marked by a standard protein (Ovalbumin, 48.1 kDa). (**B**) Kinetic curve of Pnao at 30 °C. The apparent *K*_*m*_, *kcat* and *k*_*cat*_*/K*_*m*_ values for PN at 30 °C are 0.073 ± 0.018 mM, 0.790 ± 0.074 s^−1^, 10.822 L mol^−1^ s^−1^, respectively. (**C**) Effect of pH on Pnao activity (with immediate detection). (**D**) Effect of pH on Pnao stability (with overnight incubation). The enzyme was incubated in buffers overnight. (**E**,**F**) Effects of metal salts on Pnao activity (with immediate detection) and Pnao stability (with overnight incubation). Metal salts: NaCl, NiCl_2_, BaCl_2_, CoCl_2_, CaCl_2_, CuCl_2_, MnCl_2_, ZnCl_2_, KCl, Na_2_MoO_4_, Na_2_WO_4_, CdCl_2_, BdCl_2_, FeCl_3_ and MgCl_2_. CK, without metal salts. The final concentration of metal salts was 2 mM.

A 1-mL solution containing 10 mg/mL Pnao was purified from a 3-L overnight culture of *E. coli* cells. SDS-PAGE and Superdex200 column analysis (Fig. 1A) showed that the purified Pnao has a molecular mass of approximately 54 kDa, which corresponds to the molecular mass deduced from the protein sequence. The apparent *K*_*m*_, *k*_*cat*_ and *k*_*cat*_*/K*_*m*_ values for PN at 30 °C were 0.073 ± 0.018 mM, 0.790 ± 0.074 s^−1^, 10.822 L mol^−1^ s^−1^, respectively (Fig. 1B).

### Measurement of Pnao activity

*(a) Effect of pH on Pnao activity.* Pnao showed the highest specific enzyme activity at pH 8.5 in Na_2_HPO_3_-NaH_2_PO_3_ buffer, and the enzyme activity in this buffer was much higher than that in the other tested buffers. Thus, in subsequent experiments, Pnao enzyme activity was measured in 25 mM Na_2_HPO_3_-NaH_2_PO_3_ buffer at pH 8.5 (Fig. 1C).

*(b) Effects of metal salts on Pnao activity.* Na_2_MoO_4_ and FeCl_3_ strongly inhibited the enzyme activity. In contrast, the enzyme activity was nearly twice as high in the presence of Na_2_WO_4_ as that in its absence (Fig. 1E). However, according to the ICP results, there was no difference between the protein solution (0.5938 ppb) and the control group (0.2619 ppb), indicating that Na_2_WO_4_ is not a prosthetic group. Therefore, the function of Na_2_WO_4_ during the reaction remains to be determined.

### The stability of Pnao

*(a) Effect of pH on Pnao stability.* Pnao enzyme activity was measured after incubation in buffers at 25 °C overnight. Maximal activity was observed after incubation at pH 7.5 (Fig. 1D). Thus Pnao was stored in Tris-HCl buffer pH 7.5.

*(b) Effects of metal salts on Pnao stability.* The enzyme was incubated in the solutions at 25 °C overnight. Most of the tested metal salts inhibited enzyme activity to different degrees, and Pnao showed very low activity in the presence of Na_2_MoO_4_ or FeCl_3_. In the presence of Na_2_WO_4_, Pnao showed the same activity as that reported in the activity assay (Fig. 1F).

**Figure 2:**
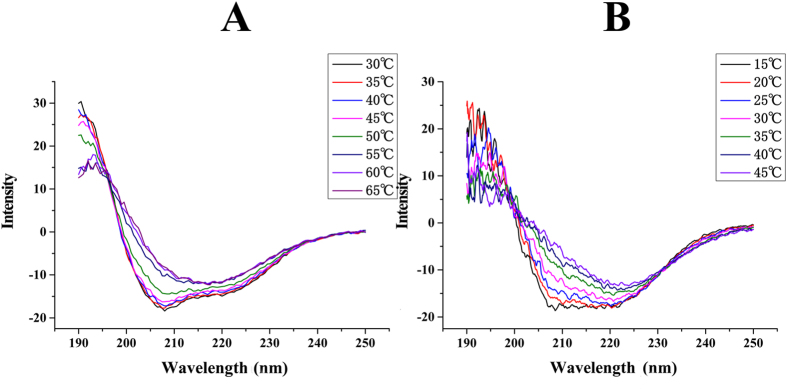
Pnao characteristics by circular dichroism spectrometer (CDS). (**A**) Spectrum of wild-type Pnao on CDS analysis. The curve started to change at temperature above 50 °C. (**B**) The spectrum of mutant Glu180Gly on CDS analysis. The curve started to change at temperature above 25 °C.

*(c) Effect of temperature on Pnao stability.* To confirm the critical degeneration temperature, Pnao stability was monitored by circular dichroism spectroscopy (CDS). The analysis showed that Pnao began to degenerate at temperatures above 45 °C (Fig. 2A). At temperatures above 50 °C, Pnao quickly denatured. However, at temperatures from 30 °C to 60 °C, Pnao enzyme activity increased (Fig. S1). Therefore, according to these results, Pnao was stable below 45 °C.

### Identification of prosthetic groups

The purified protein appeared yellow, indicating that it is bound to FAD or FMN as a cofactor. The retention time of a compound from the supernatant from a boiled protein solution in HPLC was approximately 6 min, which is similar to that of a standard solution of FAD and different from that of a standard solution of FMN. The maximum UV-Vis absorbance peaks of the supernatant were at 376 nm and 460 nm, which was the same as that of a standard solution of FAD (Fig. S2). These results indicated that FAD was the cofactor associated with Pnao. However, Pnao activity did not show an evident increase after adding additional FAD, which indicates that Pnao was already saturated with FAD.

### Site-directed mutagenesis of Pnao

**Figure 3:**
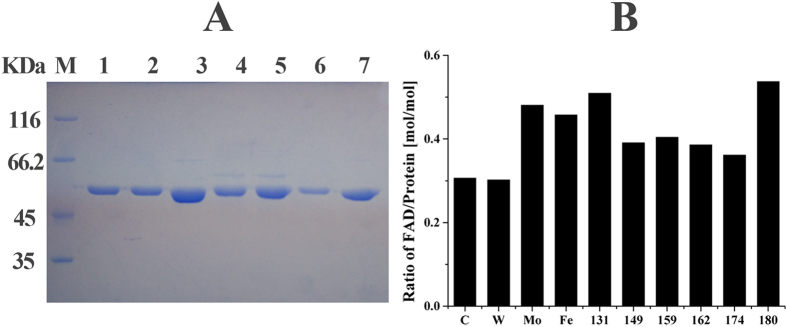
SDS-PAGE analysis of site-directed mutagenesis proteins and FAD content detection of Pnao. (**A**) SDS-PAGE analysis of purified wildtype Pnao, PnaoE131G, PnaoP149S, PnaoE159G, PnaoE162G, PnaoE174G and PnaoP180S. M, marker protein; 1, wildtype Pnao; 2, purified PnaoE131G; 3, purified PnaoP149S; 4, purified PnaoE159G; 5, purified PnaoE162G; 6 purified PnaoE174G; and 7, purified PnaoP180S. (**B**) FAD content of wildtype, mutants and Pnao with different metal salts. C, control group; W, tungsten; Mo, molybdenum; Fe, ferric iron.

Mutants of Pnao were purified by the same way of wildtype (Fig. 3A). When the enzyme activity of the mutants was assessed in the standard system at 45 °C, the Pro180Ser mutant was the only mutant that was extremely unstable. Thus, we analyzed Pro180Ser by CDS. The curve indicated that the structure of the Pro180Ser mutant changed at approximately 20 °C (Fig. 2B). Thus, we predict that Pro180Ser site is a very important residue for Pnao thermal stability.

### Reaction mechanism of Pnao

**Figure 4:**
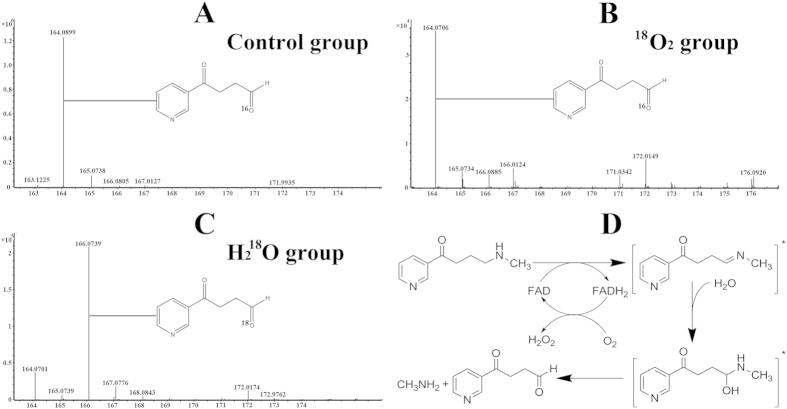
Mechanism of Pnao reaction as determined by LC-MS analysis. (**A**) The control group. A molecular peak was observed at *m*/*z* 164, which is the same as molecular weight of SAP. (**B**) The ^18^O_2_ group. A molecular peak was also observed at *m*/*z* 164, which means that O_2_ is not the source of the newly introduced oxygen atom. (**C**) The H_2_^18^O group. A molecular peak was observed at *m*/*z* 166. Thus we inferred that the newly introduced oxygen atom of succinate is derived from H_2_O not from O_2_. (**D**) A general view of the reaction process of Pnao. In the first step, PN is activated via the reduction of FAD and a carbon-nitrogen double bond is formed. Then, H_2_O attaches the carbon-nitrogen double bond and a hypothetic, extremely unstable intermediate is generated. This intermediate is transformed to SAP and CH_3_NH_2_ quickly. Finally, FAD is regenerated via the oxidation to make preparation for the next reaction.

In the LC-MS analysis, a molecular peak was observed at *m*/*z* 164 in the control, which indicated that the product was SAP. A similar peak was observed for the ^18^O_2_ group (Fig. 4A,B). In contrast, a molecular peak at *m/z* 166 and a small peak at *m*/*z* 164 were observed for the H_2_^18^O group (Fig. 4C), suggesting that the oxygen added to SAP is derived from H_2_O. We detected the products of a Pnao standard system using an H_2_O_2_ detection kit (Sangon Biotech, Shanghai, China). An evident color change was observed, indicating that H_2_O_2_ is one of the products of the reaction. When we performed the reaction in an anaerobic environment, no H_2_O_2_ was detected. These results exhibit that O_2_ and H_2_O are both involved in the reaction and that the oxygen atom in SAP originates from H_2_O.

## Discussion

The mechanism of nicotine degradation in *Pseudomonas* provides a basis for the disposal of tobacco wastes. In our previous study, we identified a novel gene in *P. putida* strain S16 whose product is involved in the transformation of PN into SAP during nicotine degradation^15^. The encoded enzyme Pnao can attack the CH-NH bond and remove the CH_3_NH_2_ group from PN. The generated intermediates are less toxic and cannot easily be transformed into carcinogenic TNSAs through nitrosation^16^. However, little is known about the characteristics of this enzyme.

Amine oxidases play important roles in the metabolism of various organisms. The amine oxidases are an enormous group of enzymes that could show variations in their features, such as thermal stability and inhibitors^17^,^18^. Thus, we performed experiments to determine the specific features of Pnao. Pnao showed thermal stability below 50 °C, which is very unique among the enzymes involved in the nicotine degradation. Thus, we analyzed the sequence of *pnao* and the encoded protein, and found that a 58-amino acid fragment displayed a higher amount of Pro-Glu residues (27.6%) than other regions. Pro and Glu residues are very important for the structural stability of proteins at high temperature^19^. Sixteen residues (Pro or Glu) in this region were changed to Ser or Gly by site-directed mutagenesis, and the mutant proteins were not stable at high temperatures. In fact, one mutant, Pro180Ser, became extremely unstable at high temperature. The absorbance of the *α*-helices and *β*-folds started to change at temperatures above 25 °C, which was very different from that observed with the wild-type protein. However, Pro180Ser was still activated and the specific enzyme activity of Pro180Ser did not decrease significantly. Thus, we predict that residue 180 is extremely important for maintaining structural stability in different thermal environments as changing this amino acid had no evident effects on the enzyme activity.

In this study, approximately 0.3 mol of FAD/mol of wild-type Pnao was obtained, which was lower than the 0.5 mol/mol of enzyme reported previously^20^,^21^. Furthermore, there was no evident loss or increase of FAD regardless of the severity of the mutation (Fig. 3B). Enzyme activity did not increase in the presence of excess FAD, suggesting that the enzyme without FAD is unable to capture FAD. When the excessive FAD was added into the culture, the FAD amount didn’t increase. These observations suggest that this uncommon FAD to enzyme ratio is not due to insufficient FAD synthesis. In pH assays, W, Mo, and Fe were proved to be functional to Pnao. Thus 1 mM W, Mo, and Fe were added into the culture, respectively; however, there was no increase or loss of the FAD content (Fig. 3B). In this case, we infer that W, Mo, and Fe do not work by affecting the content of FAD.

Some mechanistic studies of amine oxidases, which produce NH_4_^+^, described a model in which the substrate was oxidized by O_2_ to form H_2_O_2_ and a carbon-nitrogen double bond, which was subsequently hydrolyzed^22^. When we added PN to a solution containing 15 mg/mL Pnao, the yellow solution turned colorless very quickly, and then returned to yellow after all the PN was degraded. When we performed the same assay in an anaerobic environment, the solution still turned colorless; however, it never returned to yellow, which suggests that O_2_, and not PN, is involved into the oxidation of FADH_2_. In addition, we inferred that FAD was reduced first, which then activated PN to form an intermediate.

Based on our results, we believe that PN is activated via FAD reduction and that a carbon-nitrogen double bond is formed in the first step. In the next step, H_2_O binds to the carbon-nitrogen double bond, and a hypothetical, extremely unstable intermediate is generated. This intermediate is quickly transformed to SAP and CH_3_NH_2_. At last, FAD is regenerated via oxidation for the next round of catalysis (Fig. 4D).

In summary, we determined the role of H_2_O and O_2_ in the conversion of PN to SAP by Pnao and inferred the mechanism of Pnao, showing unique features, such as unsaturated FAD content, and thermal stability that is related to site-directed mutagenesis with which residue 180 is important for thermal stability. In addition, Pnao shows significant increase of enzyme activity in presence of tungstate, which is rarely found in the literature.

## Methods

### Materials

l-(–)-Nicotine (≥99% purity) was gained from Fluka Chemie GmbH (Buchs Corp., Buchs, Switzerland). Flavin adenine dinucleotide (FAD) was obtained from Sigma-Aldrich (St. Louis, MO, USA). ^18^O_2_ and H_2_^18^O were from Shanghai Research Institute of Chemical Industry. PN (98%) and 3-succinoylsemialdehyde-pyridine (SAP) were from Toronto Research Chemicals (Canada). All other reagents and solvents applied in this study were analytical grade and easily available.

### Bacterial strains, plasmids and culture conditions

The bacterial strains, vectors, and recombinant plasmids applied in this study are listed in Table 1. *P. putida* S16 and their derivatives were cultured at 30 °C in Lysogeny broth (LB) medium containing 1 g L^–1^ nicotine. *E. coli* strains were grown at 37 °C in LB medium. Kanamycin and ampicillin were used for selection at appropriate concentrations.

### Expression and purification of His_6_-Pnao *in vitro*

The full-length DNA fragment of *pnao* was amplified by PCR (primers pnaoEF-NcoI/ pnaoEF-XhoI), and inserted into the NcoI-XhoI sites of the expression vector pET28a to reconstruct pET28a-pnao. Plasmid pET28a-pnao was transferred into *E. coli* C43(DE3) for heterologous expression. *E. coli* C43(DE3) with the reconstructed plasmids were cultured in LB containing 100 mg l^−1^ ampicillin, at 37 °C to an optical density at 600_nm_ 0.8 to 1. Isopropyl-*β*-D-thiogalactopyranoside (IPTG) (200 μmol) was subsequently added to induce the culture at 16 °C for 20 h to 24 h. The harvested cells were re-suspended with 20 mM Tris-HCl (pH 7.5) buffer and disrupted by sonication in an ice-water bath. Cell debris and insoluble proteins were removed by centrifugation (12,000 × g for 30 min). The crude enzyme was loaded onto a His-bind resin. The His_6_-tagged Pnao was eluted by 200 mM imidazole after elution of the no target proteins with 20 and 50 mM imidazole. The eluted sample was loaded onto a Superdex 200 column which had been pre-equilibrated with 10 mM Tris-HCl (pH 7.5) buffer. The protein concentration was quantified by the Bradford method using bovine serum albumin as the standard. All those steps were performed at 4 °C and the collected His_6_-tagged protein Pnao was preserved at −70 °C for further study.

### Determination of the Pnao cofactor

The purified Pnao was boiled for 5 min to release the cofactor and precipitate the protein. The solution was filtered by 0.22 μm membrane after centrifugation. The filtered supernatant was detected by high-performance liquid chromatography (HPLC) (Agilent 1200 series, Hewlett-Packard Corp., Santa Clara, CA, U.S.A.) with a C-18 column (4.6 by 250 nm; 5 μm). The mobile phase was 10 mM ammonium acetate containing 30% methanol (v v^−1^). The flow rate was 0.5 mL min^–1^. The contents were monitored by determining the absorbance at 265 nm. The standard curve was drawn in the range of 0 to 0.3 mM to analyze the concentration of FAD.

### Biochemical analysis of Pnao

Buffer, Pnao and PN were all freeze-dried in advance. In the H_2_^18^O assay, powdered buffer, PN and Pnao were added into 500 μL H_2_^18^O, and the whole system was incubated at 45 °C for 1.5 h. The ^18^O_2_ labeling reaction and anaerobic assay were performed in a rubber sealed bottle attached to an anaerobic workstation (AW200SG, Electrotek Ltd, UK). All the liquid was exposed in N_2_ atmosphere for 1 h to remove the O_2_. The mixture of buffer, powdered PN and Pnao was transferred into a tube filled with ^18^O_2_ and sealed again. The reaction was performed in room temperature for 4 h.

### Enzymatic reaction *in vitro*

The Pnao activity was determined by measuring the formation of SAP on UV-2550 spectrophotometer (Shimadzu, Kyoto, Japan). SAP showed an absorption peak at 230 nm which can be measured by UV-2550 spectrophotometer. The activity could also be quantified based on the peak area of PN on HPLC. The common reaction mixture contained 2 mM PN, and 25 mM NaH_2_PO_4_-Na_2_HPO_4_ (pH 8.5) in a final volume of 0.5 mL. The reaction was started after the addition of Pnao (50 μg), and the machine would detect the slope within 30 s at 230 nm. The reaction was started after the addition of Pnao (50 μg), and ended by 1 M H_2_SO_4_ (10 μL) at 60 s.

Buffers from pH 5.0–7.5 (citric acid/sodium citrate), 7.5–8.5 (Tris-HCl), 8.5–9.0 (monosodium orthophosphate/disodium hydrogen phosphate) and 9.0–10 (sodium carbonate/sodium hydrogen carbonate) were used. The protein was incubated in buffers overnight at 25 °C for the stability assay.

In metal salt assay, NaCl, NiCl_2_, BaCl_2_, CoCl_2_, CaCl_2_, CuCl_2_, MnCl_2_, ZnCl_2_, KCl, Na_2_MoO_4_, Na_2_WO_4_, CdCl_2_, BdCl_2_, FeCl_3_ and MgCl_2_ were used to prepare the 2 mM metal solution. The protein was incubated in 2 mM metal solution overnight at 25 °C for the stability assay.

### Electrophoresis of Pnao

Sodium dodecyl sulfate-polyacrylamide gel electrophoresis (SDS-PAGE) was performed using a 12% gel in a MiniProtean III electrophoresis cell (Bio-Rad, Hemel Hempstead Corp., UK). The native page assay (Native-PAGE) was performed using a 7–12% gel in the ice-bath.

### Detection of tungsten in Pnao

Pnao was added into 30% nitrite acid solution to 10 mL and incubated at 100 °C for 1 h. The product was detected on inductively coupled plasma-atomic (optical) emission spectrometry (ICP-OES, iCAP 6000 Radial, Thermo, America).

### Site-directed mutagenesis of Pnao

The site-directed mutagenesis was performed by pEASY-Uni Seamless Cloning and Assembly Kit (TransGen, China). The primers are listed in Table 2. The mutants of *pnao* genes were inserted into pET28a and transferred into *E. coli* C43(DE3). The specific enzyme activity was detected by adding excessive FAD at 230 nm. The concentration of FAD which attached to the protein was determined by HPLC as previously described.

### Analytical techniques

PN and SAP were detected by HPLC, and the mobile phase of HPLC was a mixture of 10% methanol and 90% 1 mM H_2_SO_4_. The flow rate was 0.6 mL min^–1^, and the column (C-18, 4.6 × 250 mm) was at 30 °C. The products of enzymatic reaction were performed on an Agilent 6230 time of flight-MS equipped with ESI sources using C_18_ column (4.6 by 150 nm, 5 μm). The mobile phase was an acetonitrile-H_2_O (0.01% HCOOH [v v^–1^]; flow rate 0.4 mL min^–1^). The column temperature was at 30 °C. The samples were ionized by electrospray with a positive polarity. PN and SAP could be easily detected with thin layer chromatography (TLC). The mobile phase consists of chloroform, methanol, ethanol and H_2_O (12 : 0.8 : 6 : 0.6; [vol/vol/vol/vol]).

## Additional Information

**How to cite this article**: Hu, H. *et al.* Characterization of Pseudooxynicotine Amine Oxidase of *Pseudomonas putida* S16 that Is Crucial for Nicotine Degradation. *Sci. Rep.* **5**, 17770; doi: 10.1038/srep17770 (2015).

## Supplementary Material

Supplementary Information

## Figures and Tables

**Table 1:** Bacterial strains and plasmids used in this study.

Bacterial strain or plasmids	Characteristics
*Pseudomonas putida* S16	Ap^r^; wild type; nicotine degrader; G^–^
*Escherichia coli* strains	
DH5α	F^–^ *recA1 endA1 thi-1 supE44 relA1 deoR*△(*lacZYA-argF*)*U169 φ*80*lacZ*△M15
BL21(DE3)	F^–^ *ompT hsdS*(r_B_^–^ m_B_^–^) *gal dcm lacY1*(DE3)
C43(DE3)	F^–^ ompT hsdSB (r_B_^–^ m_B_^–^) gal dcm (DE3)
Plasmids	
pET28a(+)	Km^r^; expression vector
pET-pnao	Km^r^; NcoI-XhoI fragment containing *pnao*
pET-pnaoE131G	Km^r^; NcoI-XhoI fragment containing *pnao*E131G
pET-pnaoE159G	Km^r^; NcoI-XhoI fragment containing *pnao*E159G
pET-pnaoE162G	Km^r^; NcoI-XhoI fragment containing *pnao*E162G
pET-pnaoE174G	Km^r^; NcoI-XhoI fragment containing *pnao*E174G
pET-pnaoP141S	Km^r^; NcoI-XhoI fragment containing *pnao*P141S
pET-pnaoP156S	Km^r^; NcoI-XhoI fragment containing *pnao*P156S
pET-pnaoP180S	Km^r^; NcoI-XhoI fragment containing *pnao*P180S

**Table 2:** Primers.

Primers	Sequence (5′–3′)
Wildtype Pnao	F: ATACCATGGTGACAAAAGATGGTGATGAAGGCAGC
	R: GTGCTCGAGCGCATTGTCATTTTCTCTTTTTAG
PnaoE131G	F:ATGCACTATGGCCTAGGGGTGGAGGAGACGGT
	R:ACCGTCTCCTCCACCCCTAGGCCATAGTGCAT
PnaoE159G	F: GAGCACCTGCAGCAGGGGCGTTTGAAATTTT
	R: AAAATTTCAAACGCCCCTGCTGCAGGTGCTC
PnaoE162G	F: CAGCAGAGGCGTTTGGAATTTTTGGCTCTGC
	R: GCAGAGCCAAAAATTCCAAACGCCTCTGCTG
PnaoE174G	F: ACGAATACTACAAAGGAGCACGGAATATTTA
	R: TAAATATTCCGTGCTCCTTTGTAGTATTCG
PnaoP149S	F: GTCGGTCTTGCGAATTCAGAGACCGTCATTT
	R: AAATGACGGTCTCTGAATTCGCAAGACCGAC
PnaoP156S	F: AATGTCAAAAGAGCATCTGCAGCAGAGGCGT
	R: ACGCCTCTGCTGCAGATGCTCTTTTGACATT
PnaoP180S	F: GCACGGAATATTTATTCGCGCCCGTTTGAAC
	R: GTTCAAACGGGCGCGAATAAATATTCCGTGC
