# Supplementary material for: Characterization of Pseudooxynicotine Amine Oxidase of Pseudomonas putida S16 that Is Crucial for Nicotine Degradation
Source: Sci Rep. 2015 Dec 4;5:17770. doi: 10.1038/srep17770 (PMC4669500; doi:10.1038/srep17770)
Supplement: Supplementary Information [file srep17770-s1.doc]

**Supporting information**

**Characterization of Pseudooxynicotine Amine Oxidase of *Pseudomonas putida* S16 that Is Crucial for Nicotine Degradation**

Haiyang Hu^a, b^, Weiwei Wang^a, b^, Hongzhi Tang^a, b*^, and Ping Xu^a, b^

^a^State Key Laboratory of Microbial Metabolism, and School of Life Sciences & Biotechnology, Shanghai Jiao Tong University, Shanghai 200240, People’s Republic of China

^b^Joint International Research Laboratory of Metabolic & Developmental Sciences, Shanghai Jiao Tong University, Shanghai 200240, People’s Republic of China

^*^Corresponding author:

E-mail: [tanghongzhi@sjtu.edu.cn](mailto:tanghongzhi@sjtu.edu.cn) (Tang, H.).

Tel: +86-21-34204066; Fax: +86-21-34206647-801.

Mailing address: School of Life Sciences & Biotechnology, Shanghai Jiao Tong University, Shanghai 200240, P. R. China

**Keywords:** Nicotine, *Pseudomonas*, Amine oxidase





Fig. S1. Effects of temperature on Pnao activity. The enzyme was incubated in PBS buffer (25 mM, pH 8.5) at varying temperatures for 30 s.


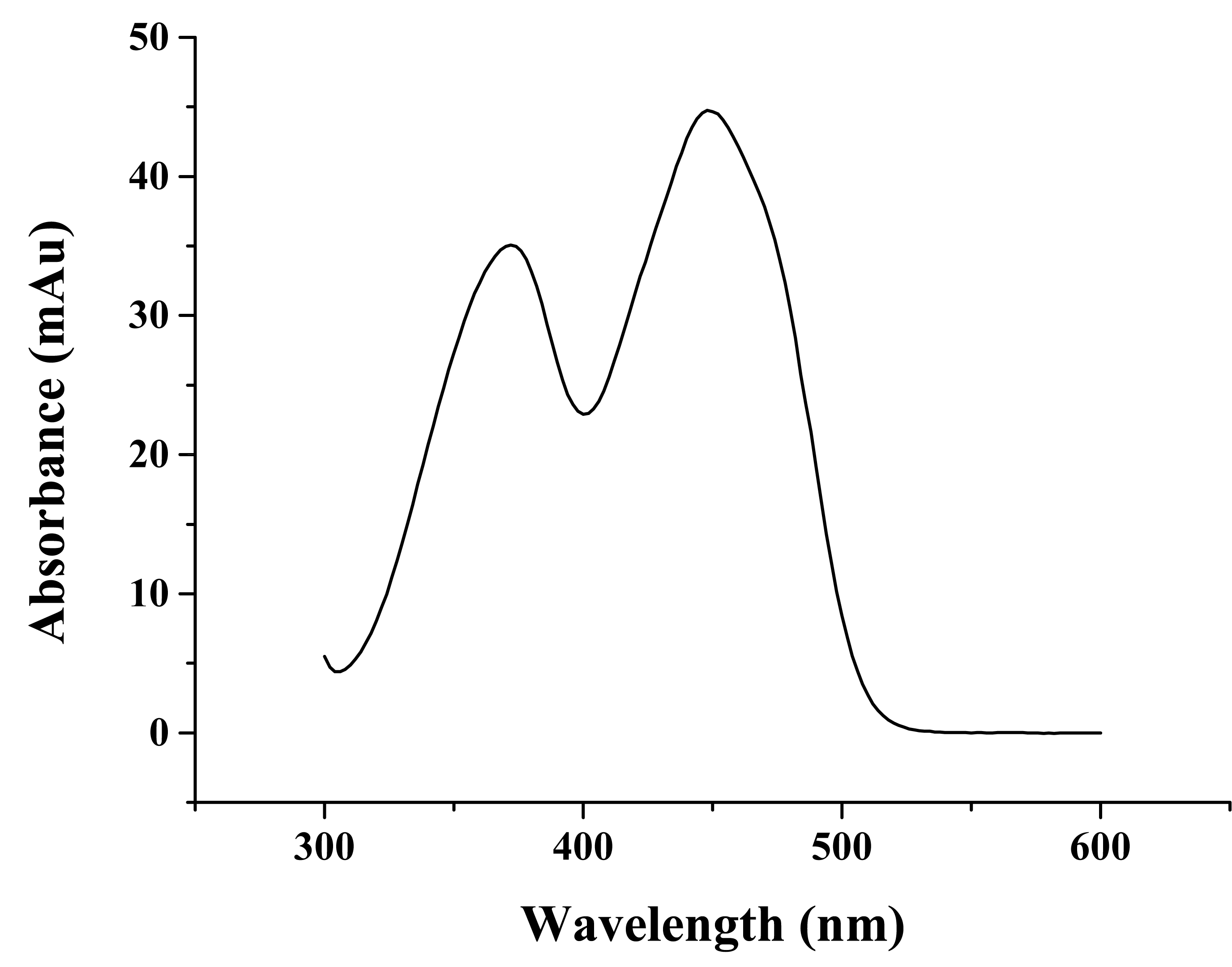


Fig. S2. The spectrum curve of FAD on HPLC. The absorbance peak of FAD is at 370 nm and 450 nm.


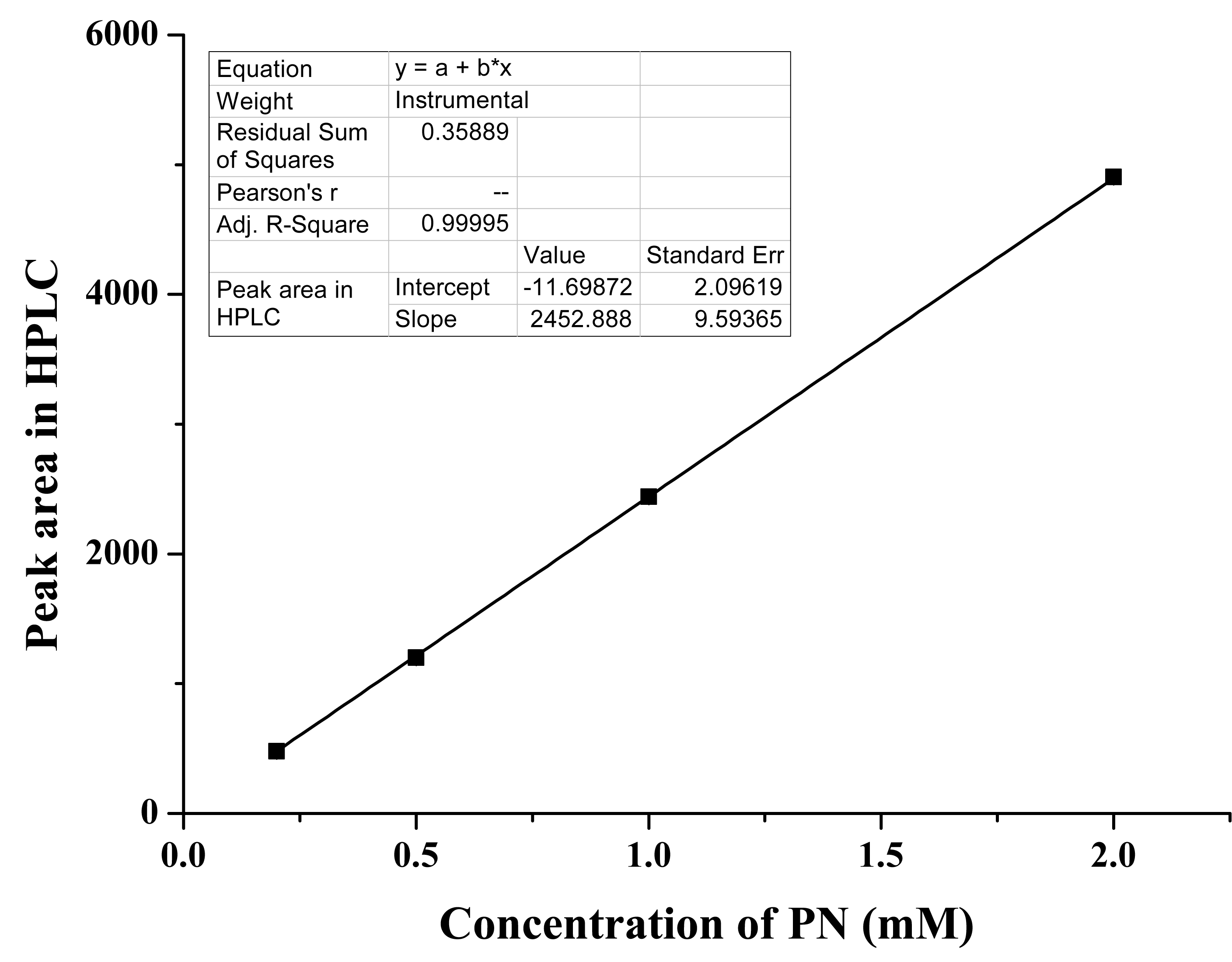


Fig. S3. The standard curve of PN on HPLC.


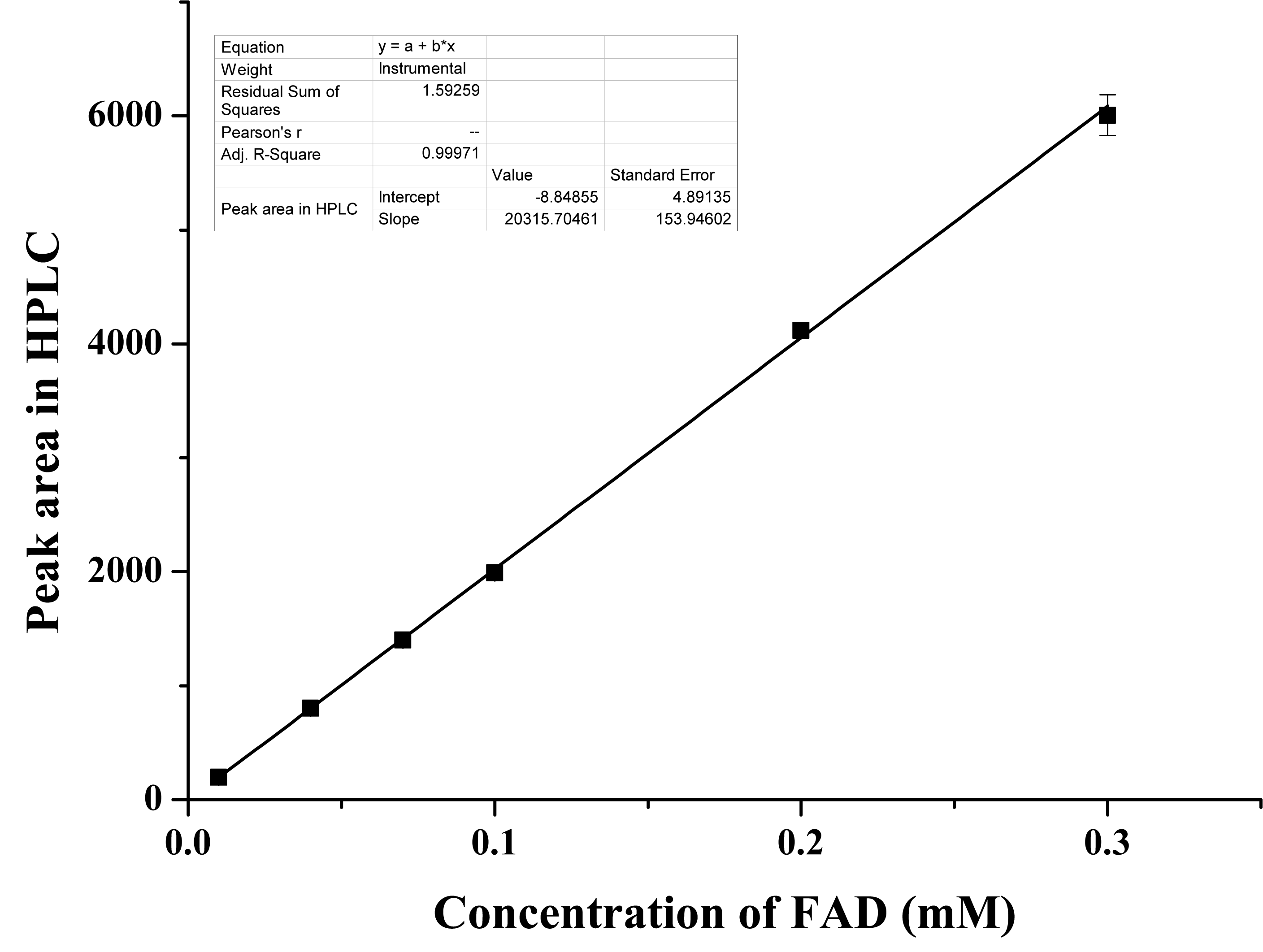


Fig. S4. The standard curve of FAD on HPLC.
